# Associating schizophrenia, long non-coding RNAs and neurostructural dynamics

**DOI:** 10.3389/fnmol.2015.00057

**Published:** 2015-09-30

**Authors:** Veronica Merelo, Dante Durand, Adam R. Lescallette, Kent E. Vrana, L. Elliot Hong, Mohammad Ali Faghihi, Alfredo Bellon

**Affiliations:** ^1^Department of Psychiatry and Behavioral Sciences, University of Miami, Miller School of MedicineMiami, FL, USA; ^2^Penn State Hershey Medical Center, Department of PharmacologyHershey, PA, USA; ^3^Penn State Hershey Medical Center, Department of PsychiatryHershey, PA, USA; ^4^Maryland Psychiatric Research Center, Department of Psychiatry, University of Maryland School of MedicineBaltimore, MD, USA; ^5^Center for Therapeutic Innovation, Department of Psychiatry and Behavioral Sciences University of Miami, Miller School of MedicineMiami, FL, USA

**Keywords:** neurite formation, brain development, dendrites, axons, dendritic spines, brain structure, epigenetics, gene

## Abstract

Several lines of evidence indicate that schizophrenia has a strong genetic component. But the exact nature and functional role of this genetic component in the pathophysiology of this mental illness remains a mystery. Long non-coding RNAs (lncRNAs) are a recently discovered family of molecules that regulate gene transcription through a variety of means. Consequently, lncRNAs could help us bring together apparent unrelated findings in schizophrenia; namely, genomic deficiencies on one side and neuroimaging, as well as postmortem results on the other. In fact, the most consistent finding in schizophrenia is decreased brain size together with enlarged ventricles. This anomaly appears to originate from shorter and less ramified dendrites and axons. But a decrease in neuronal arborizations cannot explain the complex pathophysiology of this psychotic disorder; however, dynamic changes in neuronal structure present throughout life could. It is well recognized that the structure of developing neurons is extremely plastic. This structural plasticity was thought to stop with brain development. However, breakthrough discoveries have shown that neuronal structure retains some degree of plasticity throughout life. What the neuroscientific field is still trying to understand is how these dynamic changes are regulated and lncRNAs represent promising candidates to fill this knowledge gap. Here, we present evidence that associates specific lncRNAs with schizophrenia. We then discuss the potential role of lncRNAs in neurostructural dynamics. Finally, we explain how dynamic neurostructural modifications present throughout life could, in theory, reconcile apparent unrelated findings in schizophrenia.

## Introduction

Several lines of evidence indicate that schizophrenia has a strong genetic component, including; heritability estimates of 80–85% (Cardno et al., 1999), high rates of monozygotic concordance (Cardno et al., 1999; Cardno and Gottesman, 2000) and its association with several discrete genomic regions (Sullivan et al., 2012; Ripke et al., 2013). But the role of this genetics in the pathophysiology of schizophrenia remains a mystery. Recently discovered regulatory mechanisms of gene transcription could help us bring together apparent unrelated findings in schizophrenia namely genomic deficiencies on one side and neuroimaging findings, as well as postmortem results on the other. Long non-coding RNAs (lncRNAs) are an important part of these new regulatory mechanisms as they affect gene transcription through a variety of means such as acting as Natural antisense transcripts (NATs; Magistri et al., 2012) or via dosage compensation of the XX or XY chromosomes (Moran et al., 2012). LncRNAs also regulate gene expression by a mechanism known as genomic imprinting and through its interaction with paraspeckles (Mattick et al., 2009; Wang and Chang, 2011; Moran et al., 2012). Potential lncRNA implications throughout cell biology are enormous and neurons are not excluded. Numerous lncRNAs are expressed in the central nervous system (CNS) where they participate in essential processes for normal brain development (Fenoglio et al., 2013). This is of particular importance as schizophrenia is generally considered a neurodevelopmental disorder (Marenco and Weinberger, 2000; Rapoport et al., 2005). It is therefore not surprising that there is emerging evidence of lncRNAs association with this mental illness given these molecules play crucial roles in brain development. What remains to be determined is confirmatory evidence of these findings and more importantly, which cellular process they regulate. To explore such a complex question, we have to rely on commonalities in findings from patients with schizophrenia.

Among the most consistent findings in schizophrenia are ventricular enlargement and decreased brain volume (Van Horn and McManus, 1992; Wright et al., 2000; Woods et al., 2005). How these findings come about remains under intense research, however; neuropathological studies have shown that neurons from patients with schizophrenia present shorter and less ramified dendrites and axons (Van Horn and McManus, 1992; Wright et al., 2000; Woods et al., 2005; Bellon et al., 2011). Even neuronal-like cells differentiated from skin fibroblasts exhibit shorter extensions (Brennand et al., 2011), while glial cells also from psychotic individuals exhibit abnormal arborizations (Rajkowska et al., 2002). A discreet shrinkage of dendrites and axons present throughout the brain could lead to smaller brains and enlarged ventricles, but it does not explain the complex pathophysiology of schizophrenia. However, dynamic changes in neuronal structure that are present throughout life could provide an explanation.

It is well recognized that the structure of developing neurons is extremely plastic undergoing constant extension and retraction of neuronal arborizations (Tavosanis, 2012; Urbanska et al., 2012). This structural plasticity was once thought to stop with brain development. Nonetheless, breakthrough discoveries in neurosciences have shown that the old dogma of a static neuronal structure in adulthood is no longer applicable. Instead, it is now known that neuronal structure remains plastic throughout life (Holtmaat and Svoboda, 2009; Nacher et al., 2013). Adult neurons change their shape even at baseline when they are not being stimulated by environmental cues (Mizrahi and Katz, 2003; De Paola et al., 2006; Galimberti et al., 2006; Lee et al., 2006; Nishiyama et al., 2007; Chow et al., 2009; Marik et al., 2010; Chen et al., 2011). In the presence of stimuli such as learning, these structural modifications become even more dramatic (Holtmaat and Svoboda, 2009; Caroni et al., 2012). Stress also causes drastic changes in neuronal shape including dendritic atrophy in the prefrontal cortex and hippocampus, whereas in the amygdala it promotes dendritic growth (Radley and Morrison, 2005). The adult brain’s ability to retain a degree of structural plasticity is evident. What the neuroscientific field is still trying to understand is how these dynamic changes are regulated and lncRNAs are promising candidates. Therefore, the goals of this review are to: (1) briefly present how lncRNAs exert their multiple regulatory mechanisms; (2) determine if there is evidence associating lncRNAs to schizophrenia; and (3) explore if genes regulated by lncRNAs linked to schizophrenia impact neuronal structure. We then discuss the potential role of lncRNAs in neurostructural dynamics and how dynamic structural modifications occurring throughout life could, in theory, reconcile apparent unrelated findings in schizophrenia.

## Methods

A systematic review of the literature was conducted via MEDLINE and Google Scholar using search terms such as lncRNAs, neuronal structure, dendrite, axons, dendritic spines, axonal boutons, structural plasticity, neuronal differentiation and schizophrenia. The search was limited to studies published in English. Regarding the association of lncRNAs with schizophrenia, only studies involving patients with schizophrenia were included; any articles associating lncRNAs with schizophrenia through animal models were excluded.

### Long Non-Coding RNAs Mechanisms of Action

Seventy-to-seventy five percent of the eukaryotic genome encodes transcripts that do not have an obvious potential to encode proteins and are popularly called non-coding RNA (ncRNA; Consortium, 2012; Pennisi, 2012). For many years, the role of ncRNAs remained a mystery. Only recently, significant progress has been made in understanding how ncRNAs participate in a number of essential cellular processes. NcRNAs are broadly classified into long and small ncRNA depending upon the length of the transcript. Small ncRNAs (less than 200 bp in length) include micro RNAs (miRNAs), piwi-interacting RNAs (piRNAs), endogenous small-interfering RNAs (siRNAs) and small nucleolar RNAs (snoRNAs), and have all been extensively reviewed in the literature (Carthew and Sontheimer, 2009; Ghildiyal and Zamore, 2009; Malone and Hannon, 2009; Magistri et al., 2012). On the other hand, lncRNAs are defined as transcripts longer than 200 bp. Similar to messenger RNAs (mRNA) they are RNA polymerase II transcripts that are capped, spliced and polyadenylated, yet do not function as templates for protein synthesis (Mercer et al., 2009; Ponting et al., 2009). LncRNA genes maintain features common to protein-coding genes such as promoter regions, intron-exon boundaries and alternative splicing, but lack extended translational open reading frames (ORFs). Moreover, they are mainly nuclear localized, less polyadenylated and more tissue-specific than protein-coding genes (Barry, 2014). The number of total lncRNAs is in the range of 20,000 transcripts. The crucial task is to determine whether all these transcripts are functional or not. So far, over 200 lncRNAs have been studied and many of these show evidence of functionality, at least *in vitro* (Moran et al., 2012).

Intense research during the past few years has revealed that the main function of lncRNAs is to regulate gene expression. This regulation is executed through a variety of complex mechanisms that include: acting as NATs, as well as via dosage compensation, genomic imprinting and through nuclear organization (Mattick et al., 2009; Mercer et al., 2009; Wang and Chang, 2011). In the following sections, we will briefly review each of these regulatory processes to highlight the importance of lncRNAs to maintain cellular homeostasis.

NATs are a well characterized type of lncRNA which arise from the opposite strand of protein-coding or non-protein-coding genes (Faghihi and Wahlestedt, 2009). They can regulate mRNA expression at the level of transcription via competition for regulatory factors, or through physically hindering the progress of transcription, either topologically or by being transcribed themselves (Munroe and Zhu, 2006). It is also possible for NATs to edit or activate cellular siRNA-related pathways that lead to degradation of homologous transcripts ultimately eliciting gene silencing (Kumar and Carmichael, 1998; Munroe and Zhu, 2006). Consequently, NATs can potentially regulate any step in RNA processing including translation, polyadenylation, splicing, transport or even degradation (Munroe and Zhu, 2006). NATs can also bind to epigenetic enzymes and act as scaffold to form active or repressive chromatin modifying complexes (Magistri et al., 2012). DISC-2, Evf2 and BDNF-AS are examples of NATs that have been associated with several neurodegenerative, neurodevelopment and psychiatric disorders like schizophrenia, bipolar disorder and autism (Millar et al., 2004; Williams et al., 2009; Velmeshev et al., 2013). NATs have also been documented for the Fragile X Mental Retardation gene (FMR1). Although Fragile X Syndrome (FXS) is considered a monogenetic disorder, there is evidence that supports an alternative model in which other ncRNAs contribute to FXS pathogenesis and the phenotypic variations observed among patients (Ladd et al., 2007; Khalil et al., 2008).

Dosage compensation is a hypothetical genetic regulatory mechanism that operates to equalize the phenotypic expression of characteristics determined by genes on the X chromosome so that they are equally expressed in humans carrying the XY chromosomes or the XX combination. For instance, Xist—perhaps the best studied lncRNA—is responsible for the initiation of X chromosome inactivation (Xi) in female somatic cells (Augui et al., 2011; Pontier and Gribnau, 2011). Xist silences hundreds of genes on the inactive X chromosome (Zhao et al., 2008) and this process is essential for normal brain development (Qureshi and Mehler, 2010).

LncRNAs also regulate gene expression through a mechanism known as genomic imprinting. The expression of imprinted genes depends on their parental origin and the level of differential expression of the two alleles of an imprinted gene can vary from one imprinted gene to another (Moran et al., 2012). Some lncRNAs can exert their function by recruiting epigenetic factors such as PCR2 and G9a, in order to control the imprinted expression of neighboring coding genes (Nagano et al., 2008; Zhao et al., 2008). Other lncRNAs such as Kcna1ot1, regulate genomic imprinting by interacting with repressive chromatin modifying complexes that ultimately suppress paternally inherited genes (Kanduri et al., 2006; Pandey et al., 2008). Of note, a number of authors have proposed that mental and neurological disorders such as schizophrenia and autism are often influenced by deregulation of the imprinting processes (Francks et al., 2003; Schulze et al., 2004; Schanen, 2006).

A recently discovered mechanism by which lncRNAs regulate gene expression is through their interaction with paraspeckles. Paraspeckles are membraneless subnuclear bodies (Mao et al., 2011b) that participate in nuclear organization, a phenomenon linked to genome maintenance and to the control of gene expression (Bond and Fox, 2009). These subnuclear bodies seem to regulate gene expression post-transcriptionally by retaining hyper-edited mRNAs in the nucleus (Prasanth et al., 2005; Bond and Fox, 2009; Chen and Carmichael, 2009; Clemson et al., 2009). It is noteworthy that formation and maintenance of paraspeckles requires the lncRNA named NEAT1 that localizes exclusively to paraspeckles (Clemson et al., 2009; Sunwoo et al., 2009; Mao et al., 2011a; Shevtsov and Dundr, 2011). NEAT1 has been found to be upregulated in neurological diseases such as Huntington’s disease (Johnson, 2012). Furthermore, analyses of 633 human spinal motor neurons in six cases of amyotrophic lateral sclerosis (ALS) showed that NEAT1 was upregulated during the early stage of ALS pathogenesis (Nishimoto et al., 2013).

It is clear from the preceding short overview that lncRNAs regulate gene expression through a number of complex genetic mechanisms. Deregulation of these complex genetic processes can lead to mental illnesses such as schizophrenia and therefore lncRNAs are promising candidates to explain the strong, but still uncharacterized, genetic component of this psychotic disorder. In the following section, we present data connecting lncRNAs with schizophrenia.

### Long Non-Coding RNAs (lncRNAs) and Schizophrenia

It is widely accepted that schizophrenia is a neurodevelopmental disorder (Marenco and Weinberger, 2000; Rapoport et al., 2005). It is also well recognized that schizophrenia is a severe multifactorial condition with a complex genetic component; therefore it is not surprising that previously unrecognized non-coding RNAs could participate in its pathophysiology. The lncRNAs currently associated with this mental illness are DISC-2, Gomafu, EVF-2 and BDNF-AS (Table 1). Their degree of association with schizophrenia varies from direct in cases where the actual lncRNA is abnormal in patients suffering this psychotic disorder to indirect in cases where lncRNAs regulate proteins or mRNAs known to be deficient in schizophrenia. In the following paragraphs we will discuss each of those lncRNAs with direct, as well as indirect, association with schizophrenia. We will specify the strength of the evidence linking each specific lncRNA with this psychotic disorder.

**Table 1:** **Long non-coding RNAs associated with schizophrenia**.

LncRNA	Type of lncRNA	Brain area	
DISC-2	NAT	No data currently available
Gomafu	*	CA1, hindbrain, nucleus acumbens and temporal gyrus
Evf2	NAT	Forebrain, hippocampus and dentate gyrus
BDNF-AS	NAT	Frontal cortex, infundibulum, corpus mammilare medulla, pons, cerebellum, hippocampus, amygdala, globus pallidus, putamen, caudate and thalamus

*Gomafu belongs to a novel family of noncoding RNAs refer to text for further information.

### DISC-2

In the early 2000s, a balanced translocation (1:11) (q42.1:q14.3) that segregates with schizophrenia and related disorders was found in a large Scottish family with a high loading for major mental illnesses (Millar et al., 2000; Blackwood et al., 2001). Long-term follow-up of this family has provided information about 87 family members, of whom 37 carry the translocation. Twenty nine individuals carrying the translocation, for whom psychiatric assessment was possible, have psychiatric illnesses, seven have a diagnosis of schizophrenia, one has bipolar disorder and 10 subjects present recurrent major depressive disorder. Thus, 18 of 29 translocation carriers are diagnosed with major psychiatric disorders whereas none of the 38 non-translocation carriers have any mental illness (Blackwood et al., 2001). This translocation affects the *Disrupted-in-Schizophrenia* (DISC) locus and results in a defective DISC-1 protein that lacks a carboxy-terminal (Kamiya et al., 2005; Sachs et al., 2005). Other association studies have strengthened the relationship between the DISC locus and schizophrenia. For instance, several studies support the evidence that variations at the DISC locus predispose individuals to schizophrenia, schizoaffective disorder and bipolar illness. In Hodgkinson et al. (2004) conducted a case-control study of a North American white population, they analyzed the DISC locus, describing multiple haplotypes contained within four haplotype blocks extending between exon 1 and exon 9 that are associated with schizophrenia, schizoaffective disorder, and bipolar disorder. Later, in Sachs et al. (2005) sequenced portions of DISC-1 in 28 unrelated probands with schizophrenia and six unrelated probands with schizoaffective disorder. They detected a 4 bp deletion at the extreme 3’ end of exon 12 in a proband with schizophrenia. The mutation was also present in a sibling with schizophrenia, a sibling with schizoaffective disorder, and the unaffected father. These findings support the possibility that mutations in the DISC-1 gene can increase the risk for schizophrenia and related disorders.

Expression of DISC-1 is widespread, developmentally-regulated, and high in regions of the brain that are implicated in schizophrenia, most notably the hippocampus (Millar et al., 2004). In fact, one study, using western blot analysis in postmortem brain samples, detected one major band of 70–75 kDa, which was increased in the hippocampus of patients with schizophrenia when compared to controls (Lipska et al., 2006). As human brain tissue is not easily accessible; lymphoblastoid cell lines generated directly from patients with this psychotic disorder have been used to obtain data on gene expression. In cell lines derived from members of the t(1:11) family, DISC-1 gene expression was decreased at both the mRNA and protein level in translocation carriers compared to normal karyotype controls (Millar et al., 2005).

The role of DISC-1 for proper brain function and development is not entirely understood, but is thought to be involved in neuronal migration, synaptogenesis, and glutamatergic transmission (Miyoshi et al., 2003; Morris et al., 2003; Ishizuka et al., 2006; Porteous et al., 2006). It was also recently discovered that DISC-1 can regulate dopamine signaling by interacting with activating transcription factor 4 (ATF4) and then repressing *phosphodiesterase 4D* (*PDE4D*; Soda et al., 2013). In addition, several studies have directly related DISC-1 to neurostructural dynamics, as will be presented later in this manuscript (Miyoshi et al., 2003; Ozeki et al., 2003; Kamiya et al., 2005).

While significant information is available about DISC-1, less is known about DISC-2. DISC-2 is located on the opposite strand to DISC-1 and is transcribed in a distal to proximal orientation, thus representing a NAT that lacks any significant coding potential and partially overlaps DISC-1 (Millar et al., 2000). As a NAT, it is believed to function as a negative regulator of its protein-coding counterpart, DISC-1 (Millar et al., 2000; Taylor et al., 2003; Chubb et al., 2008). In Millar et al. (2000) cloned and sequenced the breakpoints of the 1:11(q42.1:a14.3) translocation linked to schizophrenia and identified both genes DISC-1 and DISC-2. They suggested that DISC-2 presents an attractive mechanism by which DISC-1 expression may be regulated and proposed that alteration of DISC-1 and/or DISC-2 activity, by truncation and/or by abnormal regulation of expression, is causally linked to the psychiatric illnesses present in translocation carriers. Later, a systematic review compiling information about the DISC locus and mental disorders also proposed that disruption of DISC-2 might have a role in the pathophysiology of schizophrenia through dysregulation of DISC-1 expression (Chubb et al., 2008).

Although there is no direct evidence linking the DISC-2 gene to schizophrenia there are strong data supporting an association between the DISC locus and this mental illness. It must be kept in mind that most association studies do not discriminate between DISC-2 and DISC-1 in terms of positive linkage and association (Chubb et al., 2008); furthermore, many studies report markers that are within the DISC-2 gene region (Ekelund et al., 2001, 2004; Hodgkinson et al., 2004; Cannon et al., 2005). Perhaps most importantly, as mentioned above, the DISC-2 gene is also disrupted by the t(1:11) translocation (Millar et al., 2000). Consequently, there is no direct evidence yet, linking DISC-2 with schizophrenia, but its role within the DISC locus makes this association very likely.

### Gomafu

Gomafu—also known as myocardial infarction-associated transcript (MIAT) was originally identified in 2004. It was found in a specific set of neurons in the mouse retina and was described as a noncoding RNA (Blackshaw et al., 2004). In Sone et al. (2007) established Gomafu as a member of a novel family of noncoding RNAs that constitutes a cell-type-specific component of the nuclear matrix. Gomafu is located in a novel nuclear compartment, which is enriched in pre-mRNA splicing factors, where it specifically interacts with splicing factor 1 (SF1; Tsuiji et al., 2011). Indeed, Sone et al. (2007), coined the name “Gomafu” to reflect the Japanese word for the speckled pattern in which it is distributed throughout the nucleoplasm.

Gomafu is widely and abundantly expressed in the nervous system throughout development and its expression continues into adulthood (Sone et al., 2007; Tsuiji et al., 2011). In adult mice, Gomafu is found in CA1 pyramidal neurons of the hippocampus and large cortical neurons in the hindbrain (Sone et al., 2007). This family of noncoding RNA participates in retinal development (Blackshaw et al., 2004; Rapicavoli and Blackshaw, 2009; Rapicavoli et al., 2010), brain development (Mercer et al., 2010), and post-mitotic neuronal function (Sone et al., 2007; Mercer et al., 2008). Gomafu became clinically relevant, in 2006, when Albertson et al. (2006) found a transcript assumed to encode a hypothetical protein highly increased in postmortem brains of human drug abusers. Subsequent bioinformatic analyses revealed that this transcript did not encode a protein and, instead, it corresponded to Gomafu (MIAT) (Ishii et al., 2006). The same team later demonstrated that Gomafu is upregulated in the nucleus accumbens of human heroin users and suggested that dysregulation of Gomafu can influence behavior (Albertson et al., 2006; Michelhaugh et al., 2011).

Despite Gomafu’s location in a chromosomal region linked to schizophrenia (22q12.1) (Takahashi et al., 2003), it was not until 2013 that this long noncoding RNA was associated with this mental illness. An Australian team found Gomafu to be downregulated in the cortical gray matter from the superior temporal gyrus of patients with schizophrenia (Barry et al., 2014). They also demonstrated that this lncRNA regulates nuclear splicing factors that ultimately affect schizophrenia related genes such as DISC-1 and ErbB4 (Barry et al., 2014). In addition, Gomafu binds to QKI, a protein itself implicated in schizophrenia (Barry et al., 2014). The QKI gene codes for a family of alternative spliced gene products (QKI-5 kb, QKI-6 kb, QKI-7 kb and QKI-7 kb-B) that regulate myelination by Schwann cells and oligodendrocytes (McCullumsmith et al., 2007). All four QKI splicing variants are expressed in the frontal cortex of human brain and the relative mRNA expression levels of the QKI splice-variant QKI-7 kb is down-regulated in schizophrenic patients (Aberg et al., 2006). There are consequently direct and indirect data associating Gomafu with this psychotic disorder.

### Evf2

Evf2 is a NAT of the DLx5/DLx6 gene (Magistri et al., 2012). Evf2 was discovered in the developing mouse forebrain and it is transcribed from the ultra-conserved *Dlx5/6* region encoding the homeodomain transcription factors DLx5 and DLx6 (Feng et al., 2006). Dlx homeobox genes products play a crucial role in migration and differentiation of the subpallial precursor cells that give rise to various subtypes of gamma-aminobutiric acid (GABA)-expressing neurons of the forebrain, including local-circuit cortical interneurons (Poitras et al., 2010). Interneurons play a vital role in modulating the activity of the cerebral cortex and they rely on the enzyme glutamic acid decarboxylase 67 (GAD67) for the synthesis of GABA (Addington et al., 2005), the major inhibitory neurotransmitter in the brain. In Bond et al. (2009) demonstrated that Evf2 plays a critical role in regulating genes involved in the development of interneurons. This research team showed that loss of Evf2 results in imbalanced gene expression, leading to decreased GABAergic interneurons in early postnatal hippocampus and dentate gyrus (Bond et al., 2009). They also discovered that, in the developing ventral forebrain, Evf2 recruits Dlx and MECP2 transcription factors to key DNA regulatory elements in the DLx 5/6 intergenic region to control DLx5, DLx6 and GAD67 expression (Bond et al., 2009).

There is no direct evidence showing abnormal levels of Evf2 in schizophrenia. However, there are data demonstrating that GAD67 and DLx1 are defective in these patients and both GAD67 and DLx1 are directly regulated by Evf2 as mentioned earlier (Bond et al., 2009). Numerous independent research teams have found GAD67 expression to be decreased in various brain regions of patients with schizophrenia (Akbarian et al., 1995; Guidotti et al., 2000; Woo et al., 2004). Moreover, recently, Joshi et al. demonstrated for the first time that DLx1 and GAD67 mRNA levels are reduced in the orbitofrontal gray matter of individuals with this mental illness (Joshi et al., 2012). Thus, there is an indirect relationship between EVf2 and schizophrenia and more specifically between Evf2, the development of GABAergic interneurons, and this psychotic disorder.

### BDNF-AS

BDNF antisense (BDNF-AS) has been described as an endogenous non-coding antisense RNA transcribed from the human BDNF gene locus (Pruunsild et al., 2007). High levels of BDNF-AS are present in the brain, kidney, spinal cord and testis and are generated as a result of alternative splicing (Pruunsild et al., 2007). Alternatively spliced isoform diversity is common to many eukaryotic organisms and is widely used in the nervous system (Lipscombe, 2005). It is important to note that both BDNF and BDNF-AS share a common sense-antisense overlapping region (Liu et al., 2006; Pruunsild et al., 2007). More importantly, it has been demonstrated that this NAT regulates the expression of BDNF mRNA and protein *in vivo* and *in vitro* (Modarresi et al., 2012). In fact, knockdown of BDNF-AS increases BDNF mRNA levels and considerably increases the expression of BDNF protein (Modarresi et al., 2012).

BDNF belongs to a class of secreted growth factors that are essential for supporting neuronal growth, survival, synaptic plasticity and it is also involved in learning and memory neuregulin can elicit neurite outgrowth throughk (Kang and Schuman, 1995; Figurov et al., 1996; Yamada et al., 2002). While BDNF-AS has not, to our knowledge, been studied in schizophrenia, BDNF gene and protein have been largely associated with this psychotic disorder (Bellon et al., 2011). An important reduction of BDNF mRNA was observed in the prefrontal cortex and the hippocampus of patients with chronic schizophrenia (Favalli et al., 2012). In addition, a similar reduction of BDNF mRNA was observed in the dentate gyrus and hippocampus of affected individuals (Thompson Ray et al., 2011). Hence, there is only indirect evidence of BDNF-AS association with schizophrenia, as only BDNF has been studied in this context.

### Long Non-Coding RNAs, Schizophrenia and Neuronal Structure

It is well established that neurons are structurally plastic throughout development (Tavosanis, 2012; Urbanska et al., 2012). But it only recently was demonstrated that neurons retain a certain degree of structural plasticity in adulthood (Mizrahi and Katz, 2003; De Paola et al., 2006; Galimberti et al., 2006; Lee et al., 2006; Nishiyama et al., 2007; Chow et al., 2009; Holtmaat and Svoboda, 2009; Marik et al., 2010; Chen et al., 2011; Nacher et al., 2013). Moreover, this plasticity becomes even more evident during learning (Holtmaat and Svoboda, 2009; Caroni et al., 2012). The presence of a dynamic neuronal structure throughout life could help reconcile a number of apparent unrelated findings in schizophrenia as will be presented later in this manuscript. One of the factors to be established is which molecules regulate these dynamic changes in neuronal shape. LncRNAs represent promising candidates as they are prominent regulators of gene expression and they are highly active during brain development. As a first step in associating lncRNAs with variations in neuronal architecture, here we review if those genes regulated by lncRNAs linked to schizophrenia can alter neuronal structure.

**Figure 1:**
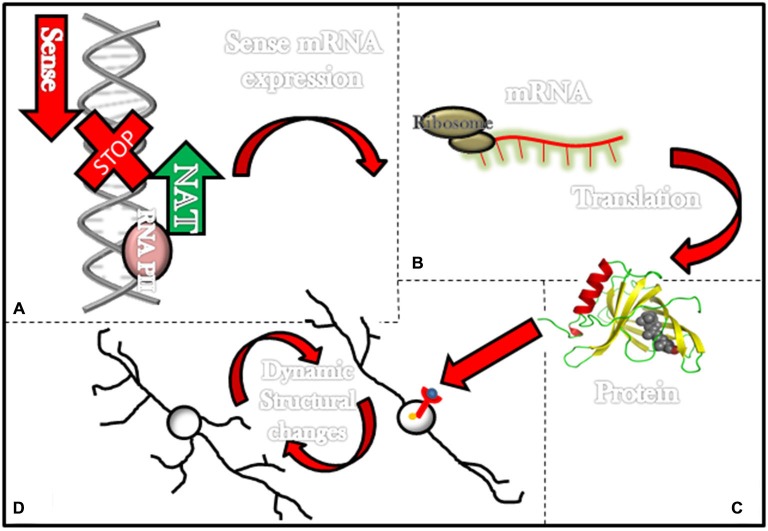
**Long non-coding RNAs (ncRNAs) associated with schizophrenia and its effects in the neuronal structure.** Most lncRNAs associated with schizophrenia are natural antisense transcripts (NATs; i.e., DISC-2, Evf2 and BDNF-AS). This figure illustrates in the upper left corner **(A)** a NAT (green arrow) being transcribed and consequently stopping transcription for genes that would have been transcribed in the normal transcription sense (red arrow). **(B)** In some instances transcription of NATs prevents the formation of mRNAs and **(C)** further translation of these mRNAs into proteins such as DISC-1 and BDNF. On the lower left corner **(D)** a diagram exemplifies how proteins such as BDNF can activate membrane receptors and then elicit dynamic structural changes in neurons.

DISC-1 has been consistently associated with neurostructural dynamics (Miyoshi et al., 2003; Ozeki et al., 2003; Kamiya et al., 2005). It has been shown that transfection of mutant DISC-1 into PC12 cells results in shorter neurites (Ozeki et al., 2003), while decreasing the expression of DISC-1 by RNA interference, inhibits neurite outgrowth in the same type of cells (Kamiya et al., 2005). Furthermore, *in utero* gene-transfer techniques in rodents have shown that DISC-1 loss induces shorter dendrites that are randomly oriented in the cerebral cortex (Ozeki et al., 2003). The mechanism of action for DISC-1 as a transformer of neuronal structure remains a work in progress. What is currently known is that DISC-1 interacts with FEZ-1 to promote cytoskeletal dynamics that lead to the extension of neuronal processes (Bloom and Horvitz, 1997; Kuroda et al., 1999). As we previously mentioned, it seems likely that DISC-2 regulates the expression of DISC-1 (Figure 1) at the DISC locus (Millar et al., 2000; Chubb et al., 2008). The fact that DISC-1 and its antisense partner DISC-2 overlap in a specific gene region and that both molecules are extensively expressed in the brain (Millar et al., 2000) during complex developmental processes and also throughout higher level cognitive tasks, suggests that there is at least some degree of interaction between DISC-1 and DISC-2 (Chubb et al., 2008).

Gomafu was recently implicated as another lncRNA involved in regulating the expression of DISC-1 and ErbB4. Overexpression of Gomafu in stem cells significantly decreases the expression of both DISC-1 and ErbB4 and their alternatively spliced variants (Barry, 2014). Conversely, knocking down Gomafu increases the expression of both DISC-1 and ErbB4 splice variants, but not their predominant, mature transcripts (Barry, 2014). Interestingly, these results almost exactly match the aberrant splicing pattern seen in post-mortem studies of patients with schizophrenia (Law et al., 2007; Nakata et al., 2009). Therefore, DISC-1 effects on neuronal structure could be regulated by either Gomafu or DISC-2.

As mentioned above, Gomafu also regulates the expression of ErbB4, a protein that has been associated with schizophrenia. Postmortem studies in patients with this mental illness showed altered levels of neuregulin-1 (NRG1) and its receptor, ErbB4, in prefrontal cortex (Chong et al., 2008). ErbB4 activation by neuregulin can elicit neurite outgrowth through two different enzymes—kinase (MAPK) and protein kinase C (PKC; Vaskovsky et al., 2000). The down-stream cascade of MAPK and PKC appears to be activation of Cdc42 which leads to fillopodium formation (Shigeta et al., 2003). Activation of Rac, also mediated by PKC, results in actin polymerization that leads to the formation of lamellipodium (Pilpel and Segal, 2004). Both fillopodium and lamellipodium are actin structures that participate in neurostructural rearrangements (Bellon, 2007). Another example of the role of ErbB4 in structural plasticity comes from a study published in 2008 that demonstrated that depletion of ErbB4 decreases the number of primary neurites in hippocampal cultures, whereas stimulation of ErbB4 using a soluble form of NRG1 results in increased dendritic arborizations via activation of the tyrosine kinase domain of ErbB4 and the phosphoinositide 3-kinase pathway (Krivosheya et al., 2008). Hence, there is clear evidence indicating that Gomafu regulates two proteins capable of transforming neuronal structure namely DISC-1 and ErbB4.

Another lncRNA, Evf2, affects neuronal shape through its association with *Dlx* genes and by regulating the expression of GAD67. In Plavicki et al. (2012) characterized the *distal-less (dll)* gene in the invertebrate nervous system; they used this single invertebrate gene as a homolog of the *Dlx* genes in vertebrates, proposing that the existence of a single *dll* gene in *Drosophila* has the potential to provide insight into how disruptions of *Dlx* function result in aberrant neuronal development in vertebrates. Altering the expression of the *Dlx* homolog, *distal-less*, disrupts normal dendrite and axon development in the olfactory system (Plavicki et al., 2012). The authors suggest that *dll* could serve as a pathfinding molecule in this brain area (Plavicki et al., 2012). Similar results are observed in rodents. Mice with a null allele of the *Dlx5* gene do not form axons that would normally connect the olfactory epithelium with the olfactory bulb (Long et al., 2003). This lack of axonal extension appears to be due to the absence of Dlx5 chemoattractant effects (Levi et al., 2003).

The influence of GAD67 on neuronal structure is of a different nature. If the enzymatic activity of GAD67 is diminished during development, either by pharmacological or genetic means, then pruning of synapses decreases (Nakayama et al., 2012). This means that elimination of the GABA synthesizing enzyme GAD67 results in more prominent arborizations. Therefore, by controlling the expression of *Dlx* genes as well as the enzyme GAD67, Evf2 can transform neuronal structure.

Finally, it is widely accepted that BDNF promotes neurostructural dynamics during development and adult life (Bellon et al., 2011). For instance, mutant juvenile mice in which BDNF levels are modestly increased display higher numbers of first-order dendrites, total dendritic length, and total branch points in dentate gyrus granule cells (Tolwani et al., 2002; Bellon et al., 2011). Similarly, the introduction of BDNF to hippocampal slices elicits an increase in the number of spines and augments apical dendritic length in pyramidal cells (Alonso et al., 2004; Bellon et al., 2011). Also, BDNF triggers dendritic spine maturation during the transition between adolescence to adulthood (An et al., 2008). Most important to our current topic is a recent association between BDNF, BDNF-AS and changes in neuronal structure. In 2012, it was found that increasing endogenous BDNF by knocking down BDNF-AS transcript results in neurite outgrowth and maturation in human and mouse cell lines (Modarresi et al., 2012). This information directly associates BDNF-AS with modifications in neuronal architecture (Figure 1).

In summary, there are data suggesting lncRNAs, associated with schizophrenia, regulate proteins that can transform neuronal shape (Figure 1). For some, such as BDNF-AS, the evidence is strong while for others, such as Gomafu and Evf2, the evidence is indirect, and the data currently available on DISC-2 remain weak.

## Discussion

After 50 years of intense research, it is now clear that a single gene or protein cannot explain such a complex illness as schizophrenia. We currently know that there are numerous genes, proteins, mRNAs, miRNAs and lncRNAs that have been associated with this mental disorder. The growing variety of molecules associated with schizophrenia could involve numerous cellular mechanisms including: cell migration, cell survival, neurogenesis, and many others. Consequently, one of the current challenges is to establish if there is a single cellular process that can consolidate all these molecules (and their functions) and, at the same time, explain the most consistent findings in schizophrenia.

We propose that a core deficit in schizophrenia is the establishment and maintenance of neuronal structure as it is now evident that the old dogma of a structural static brain is no longer valid. Recent *in vivo* evidence, using two-photon microscopy, has demonstrated that neurons remain structurally plastic throughout life (De Paola et al., 2006; Galimberti et al., 2006; Lee et al., 2006; Nishiyama et al., 2007; Chow et al., 2009; Holtmaat and Svoboda, 2009; Marik et al., 2010; Chen et al., 2011; Nacher et al., 2013) and not only during development as was originally thought. This structural plasticity involves, in addition to constant turnover of dendritic spines and axonal boutons (Holtmaat and Svoboda, 2009; Caroni et al., 2012), extension and retraction of dendritic and axonal shafts (De Paola et al., 2006; Galimberti et al., 2006; Lee et al., 2006; Nishiyama et al., 2007; Chow et al., 2009; Marik et al., 2010; Chen et al., 2011). Transformations in neuronal shape occur even when brain activity is at baseline without any particular environmental stimuli (De Paola et al., 2006; Galimberti et al., 2006; Lee et al., 2006; Nishiyama et al., 2007; Chow et al., 2009; Marik et al., 2010; Chen et al., 2011) and becomes more prominent during complex cognitive tasks such as learning (Holtmaat and Svoboda, 2009; Caroni et al., 2012). The relevance of these dynamic changes to the physiology of the brain is also apparent under stressful circumstances. Chronic stress elicits dendritic atrophy in the prefrontal cortex and hippocampus, whereas it promotes dendritic growth in the amygdala (Radley and Morrison, 2005). If the stressful event is removed, atrophic dendrites re-establish their original structure (Radley et al., 2005), which clearly illustrates how structurally plastic neurons can be. Structural plasticity is not limited to neurons. Astrocytes, after only minutes of brain stimulation, can extend processes in order to regulate synaptic transmission (Perez-Alvarez et al., 2014) and to promote excitatory synapse stability (Bernardinelli et al., 2014). Therefore, it is now obvious that continuous structural changes are part of normal brain functioning during development, as well as in adult life.

If schizophrenia is framed within the context of an ever changing neuronal shape, a number of apparent unrelated and inconsistent findings could be theoretically reconciled. For instance, it is widely accepted that schizophrenia is a developmental disorder in which exposure to famine or infection during pregnancy increases the risk for acquiring this mental disorder (MacDonald and Schulz, 2009). It has also been recognized that migrants to northern latitudes are at higher risk for developing this illness (MacDonald and Schulz, 2009). Similarly, lifetime cannabis use augments the probability of developing schizophrenia (MacDonald and Schulz, 2009). While these findings appear to be unrelated, all these risks factors may ultimately impact neuronal structure. Postmortem reports of malnourished infants have evidenced shorter and less ramified dendrites (Cordero et al., 1993; Benitez-Bribiesca et al., 1999). Similar structural deficits are observed in offspring when pregnant rodents undergo viral infection and these deficits persist even when the offspring have reached adulthood (Fatemi et al., 2002). Migration to northern latitudes is associated with vitamin D deficiency (Dealberto, 2013) and this vitamin maintains proteins directly involved in neurostructural dynamics (Féron et al., 2005). In fact, vitamin D can directly elicit growth of neuronal extensions in hippocampal neurons (Brown et al., 2003). Similarly, chronic cannabis exposure leads to altered dendritic structure in rats (Lawston et al., 2000). Unfortunately, data linking neuronal structure with all these risk factors for schizophrenia directly in patients’ cells is still lacking.

There is also information about the schizophrenia course and its clinical picture that could be explained by a defect in neurostructural dynamics. Neuroimaging studies strongly suggest that overt psychosis becomes evident while the last major pruning of brain connections is taking place (Thompson et al., 2001; Vidal et al., 2006; Sun et al., 2009a,b). This pruning normally occurs in late adolescence and early adulthood (Huttenlocher, 1990; Giedd et al., 1999; Gogtay et al., 2004; Lenroot and Giedd, 2006) when schizophrenia is most commonly diagnosed. It is possible that the removal of more connections in schizophrenia is a consequence of malformed dendrites and axons. But within this framework, how can we explain prodromal symptoms? There is no clear explanation for the origin of these symptoms; however, it is well recognized that during brain development and until adolescence the brain generates more connections than needed and only through different pruning episodes is the proper connectivity pattern finally acquired. This over-connectivity present until adolescence seems to protect patients from overt psychosis, but not from motor coordination anomalies, social difficulties and cognitive deficits (Marenco and Weinberger, 2000). These symptoms could result from neurons’ inability to constantly adjust their structure in response to external stimuli. Studies in rodents have demonstrated that learning involves drastic transformations in neuronal shape (Holtmaat and Svoboda, 2009; Caroni et al., 2012) while, in humans, different neuroimaging techniques have shown structural brain modifications after learning and training (Draganski et al., 2004, 2006; Sagi et al., 2012). One of the most consistent prodromal symptoms in schizophrenia is cognitive difficulties (Marenco and Weinberger, 2000). Consequently, a deficit in neurostructural dynamics could in theory explain the cognitive symptoms present not only during the prodromal phase but also throughout the illness course. Yet, there is still no information about brain structural deficits during learning in patients with schizophrenia.

The most consistent finding in schizophrenia, namely decreased brain volume and enlarged ventricles (Van Horn and McManus, 1992; Gur et al., 1994; Wright et al., 2000; Woods et al., 2005) can also stem from a deficit in neuronal structure. Postmortem studies have shown shorter and less ramified neuronal extensions in numerous brain areas including prefrontal cortex (Benes et al., 1986, 1991; Selemon et al., 1995, 1998; Kalus et al., 2000, 2002; Pierri et al., 2001, 2003; Broadbelt et al., 2002; Black et al., 2004), occipital cortex (Selemon et al., 1995), motor cortex (Benes et al., 1986), anterior cingulate cortex (Benes et al., 1986; Kalus et al., 2000, 2002; Chana et al., 2003), auditory cortex (Sweet et al., 2009), entorhinal cortex (Arnold et al., 1995), insular cortex (Pennington et al., 2008), subiculum (Arnold et al., 1995; Rosoklija et al., 2000), hippocampal subfields CA1, 2, 3 and 4 (Benes et al., 1991; Arnold et al., 1995; Zaidel et al., 1997; Jonsson et al., 1999; Kolomeets et al., 2005), cerebellar vermis (Tran et al., 1998), mediodorsal thalamus (Byne et al., 2002) and pulvinar (Byne et al., 2002). Even glial cells and neuronal-like cells derived from induced pluripotent stem cells have shown abnormal arborizations (Rajkowska et al., 2002). This broad range of affected brain regions strongly suggests that the entire brain is compromised.

The clinical picture of patients with schizophrenia supports this hypothesis of generalized brain dysfunction. Schizophrenia is commonly associated with psychotic symptoms; however, patients also experience negative symptoms such as lack of motivation and alogia, as well as cognitive difficulties such as attention problems and memory deficits. Less recognized, but also well documented in the scientific literature, are soft neurologic signs (Hui et al., 2009), oculomotor anomalies (Picard et al., 2008), disequilibrium (Picard et al., 2008), olfactory impediments (Turetsky et al., 2009) and the spontaneous appearance of abnormal movements even in the absence of antipsychotics (Walther and Strik, 2012). This wide variety of signs and symptoms is unlikely to originate from a single brain region or even from an isolated circuit. Instead, it could be explained by a generalized defect in neuronal structure. Within this framework, brain areas that contain neurons with more complex arborizations such as the hippocampus and the prefrontal cortex would be more affected, whereas brain regions carrying neurons with simple branches, like the olfactory bulb, would be less impaired. This proposition is consistent with the fact that the hippocampus and the prefrontal cortex are commonly associated with schizophrenia.

Based on this information, we believe there are a number of research lines suggesting a potential association between defective neurostructural dynamics and schizophrenia. One essential aspect is establishing which molecules regulate these constant changes in neuronal shape and lncRNAs are promising candidates. First, because the group of lncRNAs associated with schizophrenia is highly active during development when neuronal structure is more plastic and it is also during this life stage when schizophrenia seems to originate (Marenco and Weinberger, 2000; Rapoport et al., 2005). In addition, there is information that at least Gomafu remains active during adulthood (Albertson et al., 2006). Gomafu’s pattern of activity correlates with what is currently known about neuronal structural plasticity which is robust during development and then less active during adulthood.

A second important factor that suggests a potential association between lncRNAs, neurostructural dynamics and schizophrenia is that either lncRNAs (Table 1), or the genes they regulate, are active in numerous brain regions including: prefrontal cortex (Favalli et al., 2012), temporal gyrus (Barry et al., 2014) hippocampus, dentate gyrus (Bond et al., 2009; Thompson Ray et al., 2011; Favalli et al., 2012), nucleus accumbens (Albertson et al., 2006; Michelhaugh et al., 2011) and olfactory bulb (Long et al., 2003). This lncRNA’s capacity to act in several brain areas is in line with the clinical picture of schizophrenia and with anatomopathological results indicating there is broad brain involvement in this mental illness as was presented earlier in the discussion.

In addition, emerging information links known risk factors for schizophrenia such as malnutrition, infection during pregnancy and vitamin D deficiency with lncRNAs. For instance, the lncRNA growth arrest-specific 5 (Gas5) is implicated in certain metabolic pathways during starvation (Kino et al., 2010; Osborne-Majnik et al., 2013). It was also recently discovered that lncRNAs regulating responses to infections during pregnancy are involved with obstetric complications (Wang et al., 2014). Moreover, just last year it was revealed that vitamin D signaling can regulate the expression of certain lncRNAs (Jiang and Bikle, 2014). However, the role of these particular lncRNAs in the pathophysiology of schizophrenia needs to be further studied.

The strongest indication yet available about the possibility of lncRNAs controlling neurostructural dynamics and their potential involvement with schizophrenia comes from BDNF-AS and Gomafu. Data coming directly from patients have consistently associated BDNF with schizophrenia (Bellon et al., 2011; Thompson Ray et al., 2011; Favalli et al., 2012), while *in vitro* studies have shown that increasing endogenous BDNF by knocking down BDNF-AS transcript results in neurostructural rearrangements (Modarresi et al., 2012). But BDNF-AS has not been directly associated with schizophrenia and consequently its plausible role as a regulator of neurostructural dynamics in this mental illness has yet to be determined. In contrast, Gomafu has been directly linked with schizophrenia. This lncRNA is downregulated in the cortical gray matter of patients with this psychotic disorder (Barry et al., 2014). Moreover, Gomafu regulates genes also associated with schizophrenia (Barry et al., 2014) such as DISC-1 (Millar et al., 2000, 2005; Blackwood et al., 2001; Hodgkinson et al., 2004; Sachs et al., 2005; Lipska et al., 2006) and ErbB4 (Chong et al., 2008; Barry et al., 2014) and these genes have been shown to be directly involved in neurostructural dynamics (Bloom and Horvitz, 1997; Kuroda et al., 1999; Vaskovsky et al., 2000; Miyoshi et al., 2003; Ozeki et al., 2003; Kamiya et al., 2005; Krivosheya et al., 2008). Nonetheless, a direct deficit in neurostructural dynamics caused by either Gomafu, DISC-1 or ErbB4 in patients with schizophrenia has not been established.

In conclusion, there could be an association between schizophrenia, lncRNAs and neurostructural dynamics, but direct evidence is lacking. However, research on lncRNAs is rapidly evolving. There is tantalizing evidence that lncRNAs could help clarify the still enigmatic genetic contribution to schizophrenia. This could be particularly true if schizophrenia is approached within the context of dynamic changes in neuronal structure that start during development and continue throughout life.

## Author Contributions

Author VM managed the literature searches and wrote the first draft of the manuscript. Author DD contributed to the discussion and second draft of the manuscript. Author KEV worked on the second draft of the manuscript. Author LEH contributed to the discussion and second draft of the manuscript. Author MAF contributed in the design as well as first and second draft. Author ARL contributed in parts of the design and development of the final manuscript. Author AB designed, developed and corrected all versions of this manuscript. All authors have approved the final manuscript.

## Conflict of Interest Statement

The authors declare that the research was conducted in the absence of any commercial or financial relationships that could be construed as a potential conflict of interest.
